# Automated flow cytometry as a tool to obtain a fine-grain picture of marine prokaryote community structure along an entire oceanographic cruise

**DOI:** 10.3389/fmicb.2022.1064112

**Published:** 2023-01-06

**Authors:** Massimo C. Pernice, Josep M. Gasol

**Affiliations:** Departament de Biologia Marina i Oceanografia, Institut de Ciències del Mar-CSIC, Barcelona, Spain

**Keywords:** flow cytometry, marine prokaryotes, DNA stain, automatization, online cytometry

## Abstract

On a standard oceanographic cruise, flow cytometry data are usually collected sparsely through a bottle-based sampling and with stations separated by kilometers leading to a fragmented view of the ecosystem; to improve the resolution of the datasets produced by this technique here it is proposed the application of an automatic method of sampling and staining. The system used consists of a flow-cytometer (Accuri-C6) connected to an automated continuous sampler (OC-300) that collects samples of marine surface waters every 15 min. We tested this system for five days during a brief Mediterranean cruise with the aim of estimating the abundance, relative size and phenotypic diversity of prokaryotes. Seawater was taken by a faucet linked to an inlet pump (*ca.* 5 m depth). Once the sample was taken, the Oncyt-300 stained it and sent it to the flow cytometer. A total of 366 samples were collected, effectively achieving a fine-grained scale view of microbial community composition both through space and time. A significative positive relationship was found comparing data obtained with the automatic method and 10 samples collected from the faucet but processed with the standard protocol. Abundance values retrieved varied from 3.56·10^5^ cell mL^−1^ in the coastal area till 6.87 10^5^ cell mL^−1^ in open waters, exceptional values were reached in the harbor area where abundances peaked to 1.28 10^6^ cell mL^−1^. The measured features (abundance and size) were associated with metadata (temperature, salinity, conductivity) also taken in continuous, of which conductivity was the one that better explained the variability of abundance. A full 24 h measurement cycle was performed resulting in slightly higher median bacterial abundances values during daylight hours compared to night. Alpha diversity, calculated using computational cytometry techniques, showed a higher value in the coastal area above 41° of latitude and had a strong inverse relationship with both salinity and conductivity. This is the first time to our knowledge that the OC-300 is directly applied to the marine environment during an oceanographic cruise; due to its high-resolution, this set-up shows great potential both to cover large sampling areas, and to monitor day-night cycles *in situ*.

## Introduction

Flow cytometry is a well-established technique for the measurement of the abundance of prokaryotes, pico- and nanophytoplankton and even viruses; through this method microbial populations are discriminated based on the optical properties of the cells and/or in response to stains targeting specific cellular structures or molecules, e.g., nucleic acids (Gasol and Morán, 2015). Nevertheless, it is still a field in continuous evolution. The standard protocol for prokaryotic abundance estimation (Box 1) is simple and fast but, during an oceanographic cruise, the number of samples taken per day is limited both by the navigation time between stations and by the complexity and slowness of the CTD rosette casts; in fact, this sampling method involves the collection of seawater by deploying a set of bottles that are lowered till the desired depth and then closed and recovered on board, an operation that can last between 30 min and 1 h. The combination of these two factors gives, as a result, a low-resolution picture of the distribution of microbial communities along a surface transect. A way to improve the number of samples without increasing sampling effort is the automatization both of the sampling process and of the flow cytometry determinations.

In microbial ecology, the first attempts with automated flow cytometry were developed for phytoplankton with the use of submersible flow cytometers like FlowCytobot, CytoSub-CytoBuoy, SeaFlow (Dubelaar et al., 1999; Olson and Sosik, 2007; Thyssen et al., 2008, 2011; Swalwell et al., 2011) and the automation of the process of sampling and fixation was used for prokaryotes and picoplankton to obtain samples every 12 min which were analyzed 4 h later (Martin et al., 2005, 2008, 2010). More recently, the usefulness of automatization for the study of prokaryotes was highlighted by two papers by Besmer et al., (2014, 2016) dealing with the monitoring of microbial communities in drinking waters and freshwater environments. In this case, the entire process was automated including sampling, staining and reading of the sample signal by a flow cytometer. Based on these studies, Besmer and his team developed and now commercialize an automatic sampler (OC-300, OnCyt microbiology AG, Zurich, Switzerland) that works in tandem with a simple bench-top flow cytometer (Accuri C6, BD Accuri, San Jose CA, United States), this coupled system is the one used in the present work.

Even though the OC-300 is in the market since 2017, to our knowledge there are only three publications based on its use, two of them about monitoring microbial abundance in biofuel cultures (Haberkorn et al., 2021), and bioreactor performance (Hess et al., 2021), and the third one focus on the identification of outlying observations with machine learning (Russo et al., 2021). It seems that the potential of the OC-300 for environmental monitoring, particularly in seawater, is still unexploited.

Here we present the first application of this methodology of automatic flow cytometry directly on an oceanographic vessel for the semicontinuous assessment of abundance, relative size and biomass of prokaryotes. We evaluate its feasibility, advantages and limitations and discuss the reliability of the obtained results. In addition, the huge dataset produced by this automated system (which includes the optical properties of every single cell measured) is also perfectly tailored for computational cytometry, a set of bioinformatic tools that can define populations based on common optical properties and calculate prokaryotic community phenotypic diversity. This methodology was tested during a week-long Mediterranean cruise in early June 2021. We report here on the setup and arrangements used, and present the type of data acquired. Moreover, we highlight how this method is suited for testing hypotheses about the spatial abundance, diversity and community structure of marine prokaryotes in the surface ocean.

## Material and equipment

The system tested in this work was constituted by the OC-300 automatic sampler connected with a BD Accuri™ C6 Plus Personal Flow Cytometer. The Accuri C6 has two lasers, blue (488 nm) and red (640 nm), four filters (FICT-533 nm, PE-585 nm, PerCP-670 nm and APC-675 nm) and two scatters, Forward (FSC, 0° ± 13°) and Side (SSC, 90° ± 13°); laser power and detector voltage are fixed in this type of machine and cannot be manipulated. The flux of the machine was calibrated before the cruise and the correct functioning of lasers and filters was checked through a quality control with the appropriate beads (CS & RUO Beads, BD). This operation was also repeated after the cruise to ensure that the flow cytometer worked correctly throughout the cruise.

The reagents to reproduce this method are (i) Ultrapure or distilled water, (ii) Sodium hypochlorite solution (approx. 1% active chlorine), (iii) Sodium thiosulfate solution (Sigma-Aldrich, 72,049-250G, purum p.a. ≥98%), (iv) SYBR Green I stain (10,000X, Sigma-Aldrich, S9430), (v) TRIS buffer (Sigma-Aldrich, T6791-1 Kg, purity ≥99.9%). Other required materials are: three Pyrex bottles of 1 liter, an amber bottle for the SGI solution, a plastic or glass container for liquid waste, and 0.2 syringe filters (Thermo Fisher).

### Preparation of solutions

The total volume of the solutions is calculated based on the duration of the entire cruise, or the part of the cruise where measurements will be taken. The OC-300 needs *ca.* 300 mL per day for cleaning operation and, as a general rule, one liter of each of the cleaning solutions (sodium thiosulfate, sodium hypochlorite and ultrapure water) has to be prepared for a 3 days cruise. It is worth to mention that these reagents dissolve quickly in ultrapure water and, in case it is not feasible to transport ready-made solutions to the boat, it is suggested to weight the exact amount of sodium thiosulfate and TRIS at the home laboratory and prepare the final solutions directly on the ship. For the solution of TRIS buffer and sodium thiosulfate, which is the dilution media for the SGI stain, the volume is 125 mL since it has to be changed every 7–10 days to ensure the correct working of the stain.

#### Sodium hypochlorite solution

Dilute 300 mL of commercial bleach, which contains around 3% of active chlorine, with 600 mL of ultrapure water to obtain a final solution with *ca.* 1% of active chlorine. Filter the solution through a 0.2 μm filter. If it has to be prepared on the ship, it will be useful to bring 60 mL plastic syringes with sterile syringe filters. Store at Room Temperature (RT).

#### Sodium thiosulfate (100 mM)

Mix 15.8 g of sodium thiosulfate powder in 1 L of ultrapure water in a glass bottle, shake the mix until all powder is dissolved and autoclave it. For a long cruise, bring 1 L bottle for each 3 days, plus an extra one. This solution can be stored at RT.

#### TRIS buffer (10 mM, pH 8) with sodium thiosulfate (50 mM)

Add 1.2 g of TRIS base and 7.9 g of sodium thiosulfate to 900 mL of ultrapure water. Measure and adjust pH to 8.0 by adding HCl. Autoclave and store it in the fridge.

#### SYBR green I solution

Filter 125 mL of solution number 3 through a 0.2 Swinnex filter and deposit it into an amber bottle, then add 25 μL of SYBR Green I (SGI) stock solution, mix well (Final concentration 2X). Prepare this solution right before use. This solution lasts for 7–10 days at RT.

## Materials and methods

The objective of this procedure is to improve the resolution of standard flow-cytometry sampling for surface heterotrophic prokaryotes during an oceanographic cruise. In order to validate this procedure, we tested the automatic sampler directly on a ship to (1) compare with values of abundance obtained with the standard protocol, (2) identify possible issues connected with the ship’s environment.

### A brief description of the OC-300 functioning

**Figure 1:**
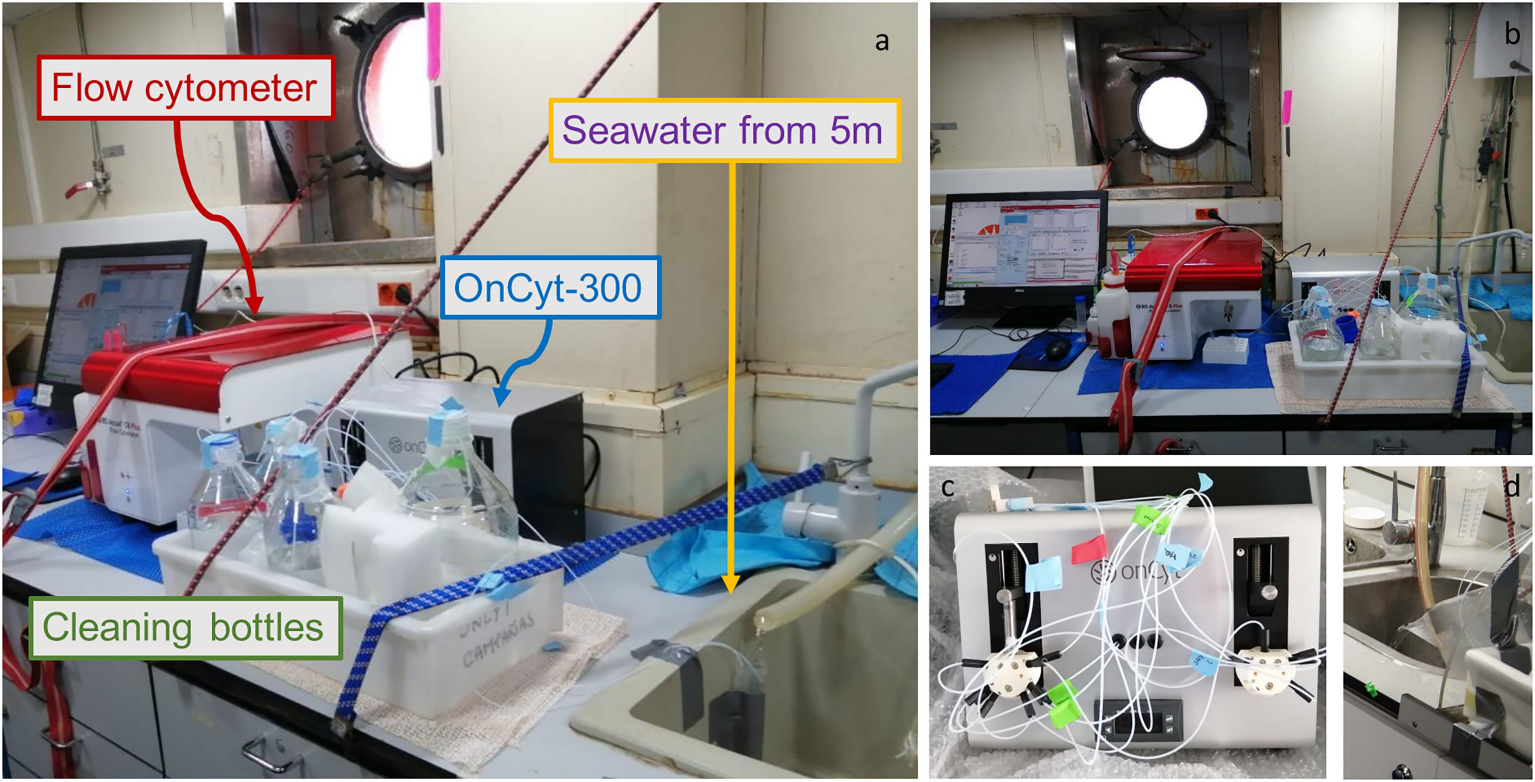
Photos of the system set-up, **(A)** Lateral view of the system with a falcon as a water collecting system from the pump. The little white tubes bring water directly to the OC-300 (grey box behind the cleaning bottles) that then sends the sample to the flow cytometer (red lid machine) **(B)** Frontal view of the system **(C)** OC-300 tubes preparation **(D)** Bucket as alternative water collector.

The OC-300 is an automatic sampler which was first developed to work in conjunction with a flow cytometer of the Becton Dickinson Accuri type, although the developers informed us recently about the possibility to connect the machine to a flow cytometer belonging to other brands (e.g., Beckman Coulter Cytoflex). Once the sampler is connected to the flow cytometer (Figure 1), all operations are mediated by a software (cyON) that takes control over the Accuri’s regular software and works based on python scripts. In a normal routine, and after the initial cleaning, the system uses a glass syringe to take both the sample and the stain, mix them (1:1) and incubate them in a chamber for 10 min at 37°C. After that, the syringe mediated again the movement of the sample from the incubation chamber to the flow cytometer in order to be analyzed. Sample run for 1 min at fast speed (66 μL min^−1^), and then a cleanup round is imposed. Other procedures involving several staining steps can be applied (e.g., NADS staining, which involve the use of Propidium Iodide at the same time of SGI). Specific characteristics, such as the pacing of the sampling, the duration and temperature of the incubation, the number of sampling tubes involved and the number of stains used, can be set by manipulating the python scripts. A minimum time of 15 min between samples is mandatory due to the cleaning operation; the machine cleans the tubing system, the syringe and the mix chamber with sodium thiosulfate (which is a quencher of SGI), sodium hypochlorite, and ultrapure water. The machine allows a maximum of 11 different samplings tubes (i.e., 11 samples can be taken at each run) but for environmental measures one tube is enough; nevertheless, considering the duration of the cruise, it was preferred to sample seawater with 3 tubes that took a sample in succession every 15 min, since in the event that one or two tubes get clogged, without operator notice, at least one measurement would be obtained every 45 min. There is a distance limitation between the automatic sampler and the source of water based on the maximum length of the tubing (120 cm is the max distance tested by the developers).

### Setting of the system

Samples are obtained from a faucet connected with the ship’s intake pump which samples water at 5 m. Submerged-pump systems are generally present on a normal research vessel. The water was let run for around 30 min before starting. The continuous flow of seawater fell directly from the faucet into a 1 l bucket which was tilted to let the water pour over. Three sampling tubes were taped to the bucket in order to allow the collection of the circulating water near the surface and avoid to take the relatively still water toward the bottom of the bucket. It is important to fix the bucket so that the water keeps being continuously renewed. An alternative, as shown in Figure 1 is to direct the flow to a falcon tube and sample water from the top of it, in this case it is fundamental to build a system resembling a half funnel to compensate for the change of the position of the water flow due to ship movement (Supplementary Figure S1). Once the sampling tube is taped to the bucket/falcon, it is essential to measure the length of the sampling tube and incorporate that value into the script for a correct functioning of the sampler, since the total volume incorporated depends on the length of the tube. With a longer tube, more pressure will be needed to deliver the right amount of liquid into the syringe.

Considering the daily need to refill with cleaning reagents, it is useful to close the bottles with parafilm, fixing the tubes to the bottles with tape. For the SGI a hole was drilled in the middle of the amber bottle lid. All the bottles were placed together in a box, well subjected and protected from movement and potential breaking. Special care was taken with the amber glass bottle containing SGI which is a known mutagenic substance (Bourzac et al., 2003). The entire system occupies a minimum surface of around 1 m^2^ placing the computer on top of the Accuri-C6.

### Procedures

Table 1 lists a series of steps and tips important for the functioning of automatic flow cytometer on a ship before, during, and after the cruise with the goal of facilitating system operations, from sampling to data management. When the cyOn software takes over the Accuri, a predefined template is automatically opened to visualize the measuring of events for each sample and check the effectiveness of the cleaning steps through the absence of events in the cytograms when ultrapure water was running. The template establishes the threshold of data collection (in this case 800 in green fluorescence, FL1), the speed of reading (fast) and a gate for the population of interest in a cytogram of green versus red fluorescence (see Gasol and Morán, 2015), which separates cyanobacteria from heterotrophic bacteria. Histograms of green fluorescence versus counts, and green fluorescence over time are also displayed in the template. Since only one stain was used, a normal practice for the abundance quantification protocol with marine microbial samples, no quenching depletion was expected and, for the same reason, no compensation was applied to correct for spillover. SGI does have spillover over the red channel (FL3), which leads to a visualization of heterotrophic bacteria as a diagonal population in a cytogram of green versus red fluorescence, but this normally does not interfere with the gating of the autotrophic population (Supplementary Figure S2). Once the cruise was completed, the total prokaryote community was manually gated for all cytograms of side scatter (SSC) versus green fluorescence (FITC) using the second software associated with the OC-300 named cyPlot. The adequacy of the gate was visually checked for the entire dataset, and the number of events per sample extracted as an excel table. Abundance then was calculated from gated events μL^−1^ times 2 (because of the dilution with SGI is 1:1), and then times 1,000 to obtain cells mL^−1^. The raw cytograms of this cruise (*n* = 366) are deposited in the SEANOE repository (seanoe.org) and available through this link https://doi.org/10.17882/90671. Abiotic parameters (Temperature, Salinity and Conductivity) of the surface water were collected in continuous mode along the entire cruise by a SBE 21 SeaCAT Thermosalinograph (Sea-Bird Scientific, United States).

**Table 1:** Key steps of the procedure.

Before the cruise
Prepare the solutions as described in the protocol
Prepare scissors, ruler and tape for make new tubes on boat
Set parameters (e.g., fluorescence threshold) in the Accuri C6 template
Check the functioning of the OC-300 and of the Flow-cytometer
Check the presence of ultrapure water machine on boat
Check the access to a continuous seawater pump
Check the space near the seawater pump (at least 1 square meter)
During the cruise
Check the correct functioning of Flow Cytometer
Set up the system (Accuri C6 + Computer + OC-300 + Bottles)
Fill the cleaning bottles with their respective solutions
Be sure that the sampling tubes are less than 120 cm
Change the volume in the script according to the sample tube length
Start the initializing procedure
Start to measure
Refill everyday till 1 L the cleaning solutions
At the end clean the system
After the cruise
**For abundance**
Open the cytogram with cyPlot
Gate the desired population
Collect events, average and mean of the fluorescent channels
**For computational cytometry application**
Extract .fcs files
Rename the files and group it in one folder
Use FlowCore to: Visualize, transform, normalize, gates and subset your samples
Use PhenoFlow to calculate phenotypic diversity
Use FlowSOM to identify different groups

### Statistical analysis and computational analysis

Regression analysis was used to compare between regular flow cytometry protocol and the automatic method whereas the correlation between the prokaryotic abundance and the abiotic parameters was tested with a Pearson correlation coefficient (both performed with Rstudio). The computational analysis required the cytograms of the entire sampling period to be pooled together, in this regard it is important to highlight that, although the cyON software generated folders named with a time signature (e.g., 2021-06-03_11-01-38), the files contained in each folder are always named with the same alphanumeric code (e.g., A04.fcs, A08.fcs and so on). For the present analysis each file was renamed as foldername_filename.fcs (e.g., 2021-06-03_11-01-38_A04.fcs). The python script used for this task is presented as Supplementary material.

Cytograms were visualized with the flowCore R package (Hahne et al., 2009), first the fluorescence values were transformed (using the arcsin function) for better visualization, in a second step the events of interest were manually gated in a cytogram of forward scatter versus green fluorescence to delete noise, and finally all cytograms were normalized against the maximum green fluorescence being the normalization a fundamental step for successive fingerprint analysis (Props et al., 2016). In order to allow diversity comparisons and also to reduce computational time, the number of cells was subsampled to the minimum number of cells retrieved among our samples (in this case, 10,303).

For the definition of clusters, groups of cells with similar values of fluorescence and scatter were grouped in 100 clusters and were identified with R package FlowSOM (Van Gassen et al., 2015). These clusters were grouped again to simplify the analyzes in 10 entities named metacluster.

## Results

### Comparison of the OnCyt-300 method versus flow cytometry standard protocol

A general stain used for acid nucleic in bacteria is SGI which is usually diluted in DMSO and unfrozen the same day of the measurement. SGI is chemically very stable and allows for several cycles of freezing/unfreezing without evident changes in its efficiency. With the automatic sampler, although the stain used is the same, the conditions are quite different. In this method SGI is dissolved in a solution of TRIS plus sodium thiosulfate and kept at room temperature for 7–10 days. The efficiency of this SGI solution was tested by the developer company (Besmer et al., 2014) and will be discussed in the following paragraphs.

The developers found a very good relationship (*R*^2^ of 0.99) when comparing unfixed samples enumerated with automatic and not automatic methods (personal communication). Since the automatic sampling does not include a fixation step, and knowing from the literature (Bullock, 1984) that fixed samples tend to stain better due to cell wall permeabilization caused by fixation, allowing an easier penetration of the stain inside the microbes, it was decided to test the similarities between our usual protocol (Box 1, fixation with a mix of paraformaldehyde and glutaraldehyde, incubation at room temperature, Gasol and Morán, 2015) against the OC-300 protocol (no fixation, incubation at 37°C). For the usual protocol, water samples were taken from the bucket with a pipette near the area where the OC-300 tubes were taped and, as much as possible, at the same time of the automatic sampling. A total of 10 samples were collected. A significant positive relationship was observed between the two methods (*n* = 10, *R*^2^ = 0.30, *p* = 0.0472, slope = 1.01), with the OC-300 protocol giving slightly higher values (average 20% more). This difference can possibly be explained by likely errors in the manual sampling, which has to be done at the same time as the automatic machine for this comparison, and by the incubation at 37°C, in addition to the fixation itself.

### Temporal and spatial variations of prokaryotic abundance

**Figure 2:**
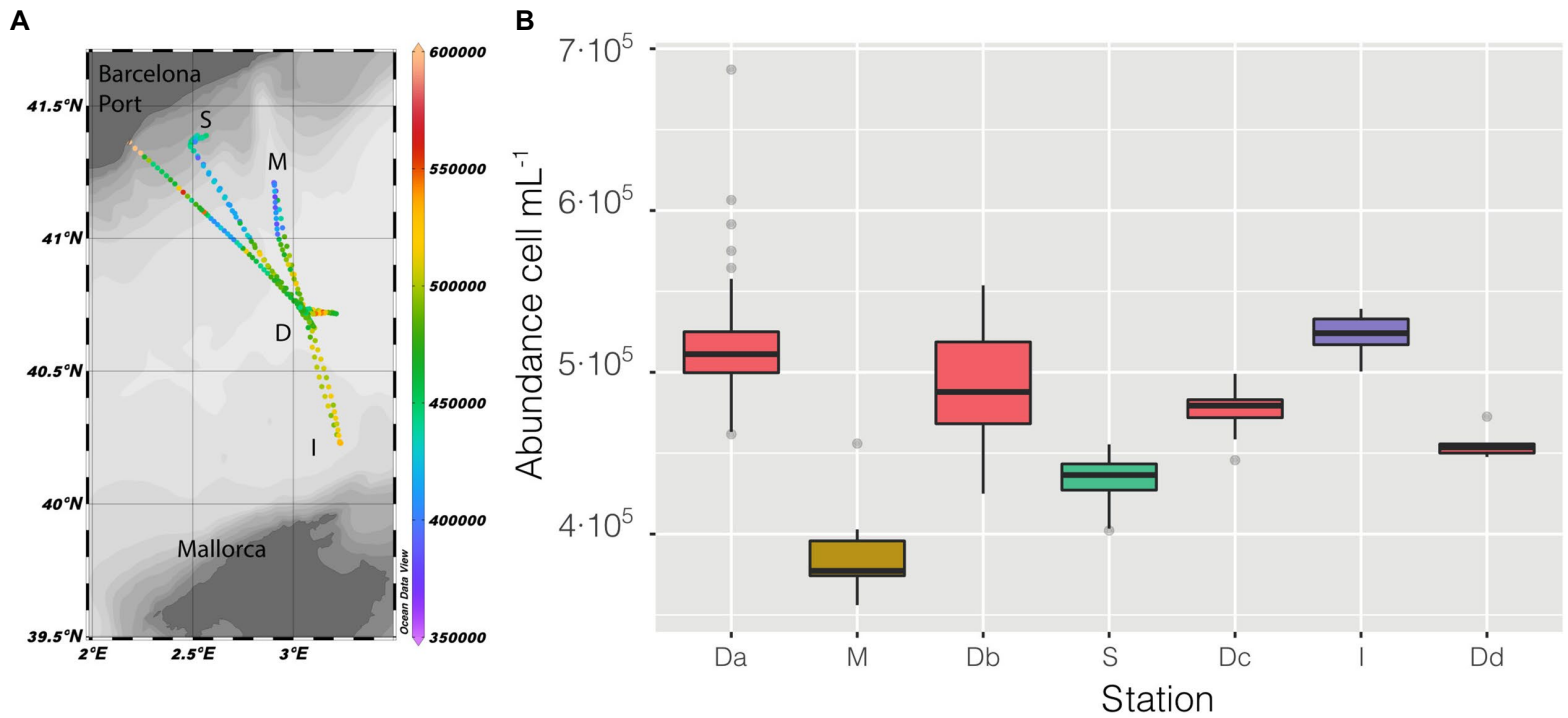
**(A)** Map of the cruise, with prokaryotic abundance (cell mL^−1^) along the entire cruise presented in a color scale, the depth of the stations is also presented as shaded. **(B)** distribution of prokaryotic abundance, presented as a boxplot for each station, the time of permanence in each station varies (Da = 21 h, M = 2 h, Db = 6 h, S = 9 h, Dc = 7 h, I = 3 h, Dd = 1 h).

Sampling started at station D (Figure 2A), and followed a kind of flower shape going out from the center and back to it, twice toward the coast (stations M and S) and once between the islands of Mallorca and Minorca (Station I), the vessel stopped at the center four times (named in Figure 2B as Da, Db, Dc, Dd) and the measurements lasted until the moment it made landfall in the Barcelona Harbor.

**Figure 3:**
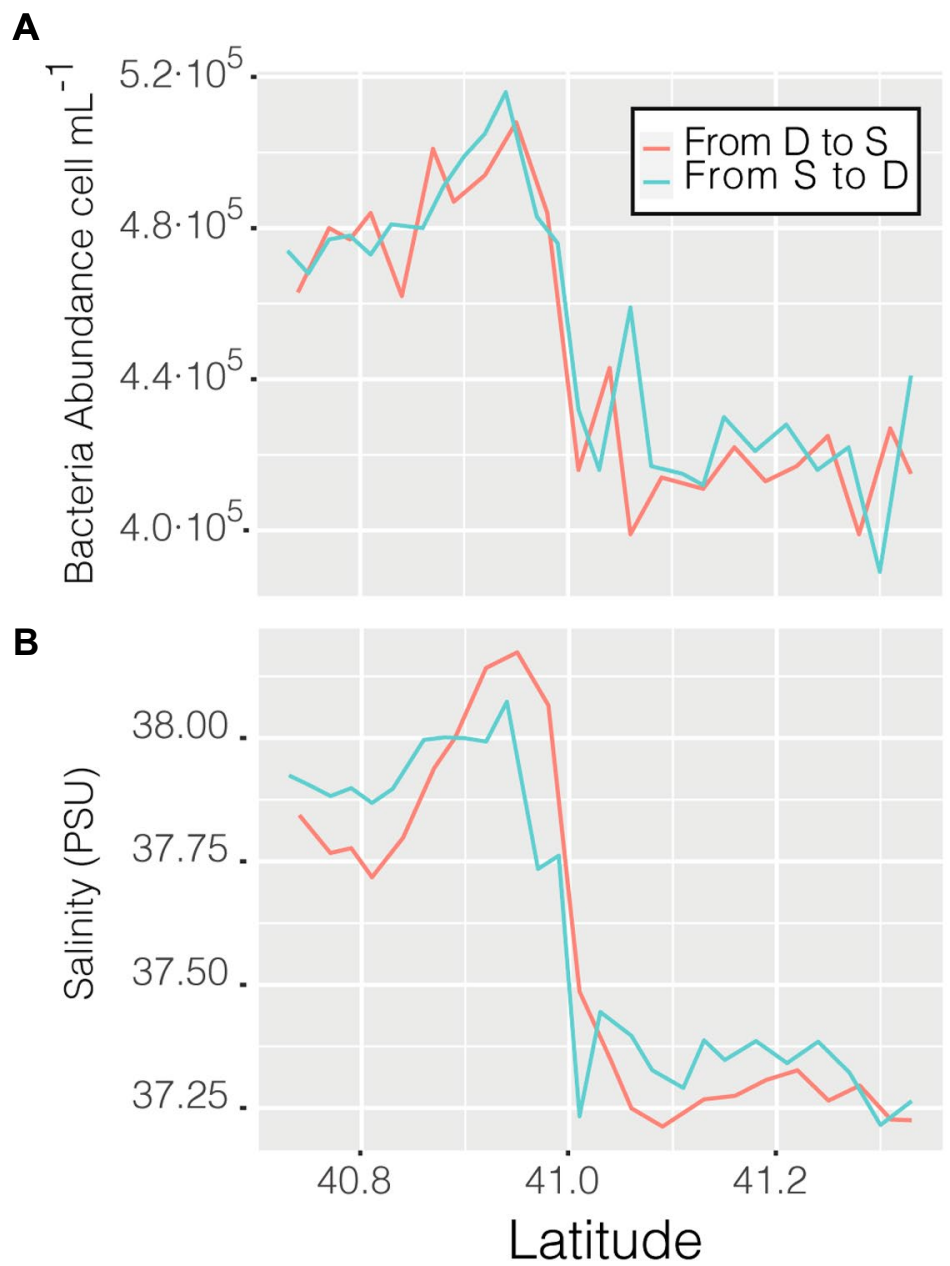
Prokaryotic abundance along the transect between station D and S **(A)** and salinity (psu) of the same transits **(B)**.

Abundance varied through space and time from a minimum of 3.56 ·10^5^ cell mL^−1^ in the coastal station M till a maximum of 6.87 · 10^5^ cell mL^−1^ in the clear waters of station D, this excepting the last 3 points when entering the Barcelona port, where abundances peaked to 1.28 · 10^6^ cell mL^−1^. Of the abiotic variables measured, conductivity was the one that better explained the variability of abundance (*n* = 366, *R* = 0.36, *p* < 0.001) but, when not considering the harbor area (the 3 final values), the *R* increased to 0.60. The correlation of abundance with temperature and salinity were *R* = 0.51 and *R* = 0.38, respectively, (for both *n* = 363, *p* < 0.001). Excluding the extreme values found in the harbor area, the values of prokaryotic abundances decreased going from station D to more coastal areas (M and S, Figure 2B), while they increased when sailing South-West toward the islands. Considering only station D (Figure 2 boxplot, red squares), the median value of prokaryotic abundance decreased over time from day 1 to day 4. This trend could easily have been overlooked with Niskin-based sampling since we would have had a single value instead of a median of multiple values for the same point. Interestingly, comparing the transits back and forth from station D to station S (Figure 3A), the abundance patterns showed a strong similarity, this is particularly important considering that the ship spent 9 h at station S. A similar situation was found comparing the transects back and forth from station D to M (data not shown). It appeared that different water masses had a stable bacterial abundance and that there was a marked change at *ca.* 41° of latitude, the change of water conditions is evidenced by the change in salinity (Figure 3B).

The observed variations in abundance along the cruise could be due by a progressive weakening of the stain or could be a true response of the prokaryotic community to changes in the environment. It is true that considering only station D, the abundance was better correlated with time (*n* = 149, *R* = −0.59, *p* < 0.001) than with conductivity (*n* = 149, *R* = 0.47, *p* < 0.001) but, considering the entire cruise (*n* = 366), time did not have a significant correlation with abundance (*p* = 0.4332) while conductivity (an environmental factor) became significant (*R* = 0.36, *p* < 0.001). If we do not consider the extreme high values of abundance found in the harbor, the correlation with conductivity (*n* = 363, *R* = 0.59, *p* < 0.001) is even stronger than the one with time (*n* = 363, *R* = −0.36, *p* < 0.001). This led us to think that differences in abundance are determined by the environmental difference and not by the weakening of the stain.

**Figure 4:**
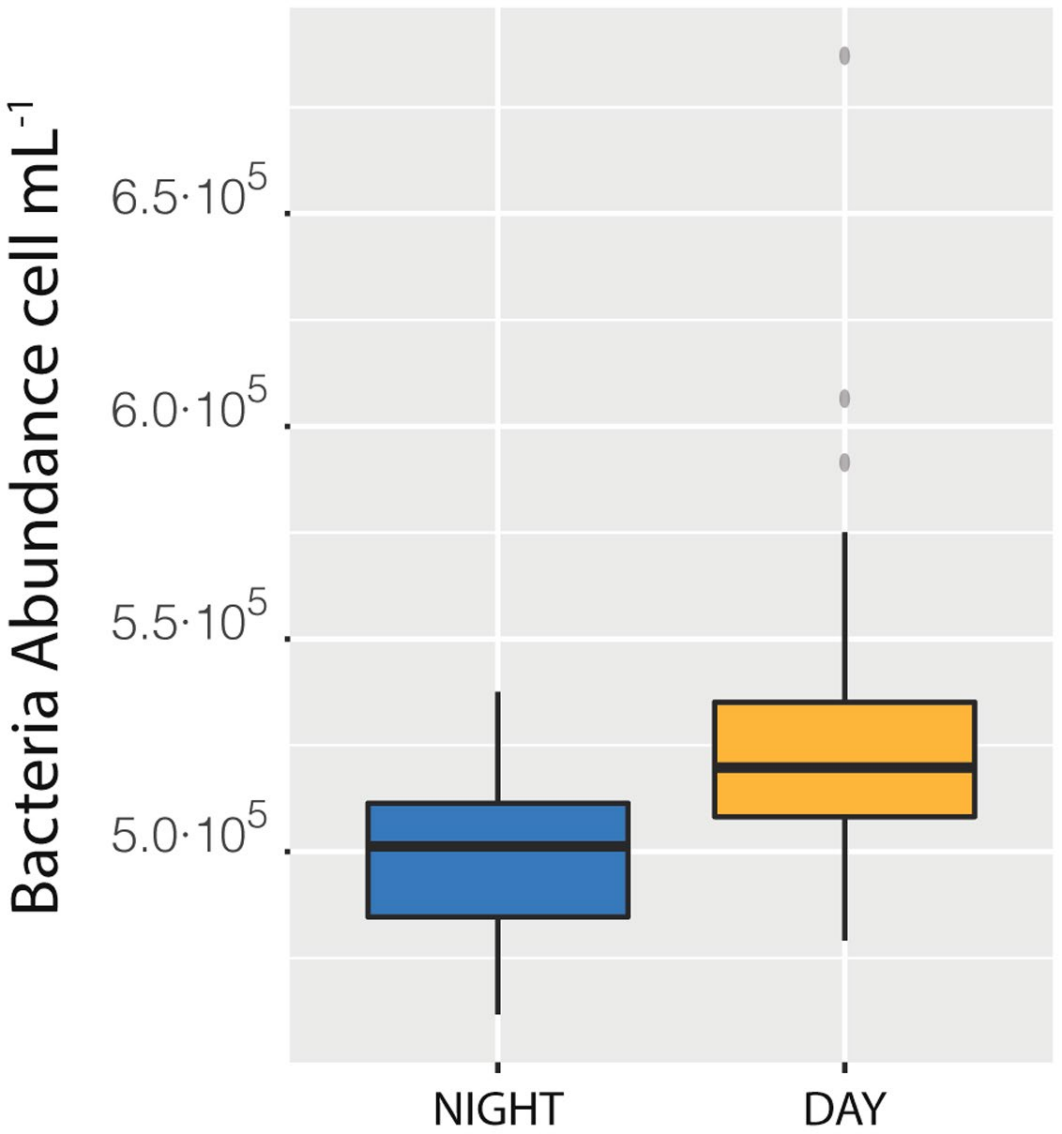
Difference between the median of prokaryotic abundance of the samples collected during the day as compared with sample collected during the night in station Da.

We also measured a full 24 h cycle (85 samples) in station D. The median of bacterial abundances during the daylight hours (Median = 5.20·10^5^ cells mL^−1^) was higher than the median of the samples taken during the night (Median = 5.01·10^5^ cells mL^−1^, with a difference of 0.18·10^5^, that is 3.6%, Figure 4), a difference statistically supported (*t*-student *p* < 0.0001). This day-night effect disappeared when considering the entire cruise, indicating that the daily variability was lower than the spatial one. The net growth rate, calculated as the slope of the natural logarithm of abundance versus time was 0.04 ± 0.01 which would indicate a duplication time of 18 days, quite reasonable (see compilation in Kirchman, 2016).

### Storm and movement of the ship

In the early hours of June 5th, a medium-intensity storm occurred during the transit from station D to S. As it can be observed in the variations of prokaryotic abundance over time (Supplementary Figure S3), the storm did not seem to affect the measurements: the abundance values were in the same range as in the hours before the storm and, as shown before in Figure 3, they closely resembled the values of the day after the storm in a similar geographic position. The lack of effect of (reasonable) ship movement on machine performance was also observed and confirmed numerically on a successive cruise where ship movement data (pitch and roll) were available (Supplementary Figure S4). Variation of prokaryotic abundance seems independent from the movement of roll. When the ship was still, it was found from the lowest to the highest value, whereas when the roll was higher a narrow range of values was observed and this movement was not correlated with abundance. The progressive narrowing of the range of abundances can be easily explained by a greater homogenization of the waters as movement increases. Actually, the abundance values corresponding to periods of high roll motion were in the range of most other data points, in other words, the abundance measurements appeared to be independent of ship movement.

### Relative size and biomass

Forward scatter (FSC) after illumination with, e.g., a blue laser can be taken as a relative measure of size (Allman et al., 1990; Gasol and Del Giorgio, 2000). An advantage of the automatic sampler is that its gating software (cyPlot) allows easy extraction of the mean and the median intensity of all variables, including FCS, for each sample point of the entire cruise. In the case study, higher average cell sizes were observed near the coast compared to stations D and I (Supplementary Figure S5), and were generally inversely proportional to abundance: excluding the 3 points in the harbor, where size and abundance changed abruptly, the Pearson correlation between mean relative size per sample and abundance was significant with a moderate inverse relationship (*n* = 363, *R* = −0.57, *p* < 0.001). Relative size was also negatively correlated with several environmental variables including temperature (*n* = 363, *R* = −0.52, *p* < 0.001), salinity (*n* = 363, *R* = −0.55, *p* < 0.001) and conductivity (*n* = 363, *R* = −0.67, *p* < 0.001). An approximation of the actual bacterial size could be made by calibrating with the FCS of beads of known size (0.5 μm Fluoresbrita Multifluorescent Microsphere by Polysciences Inc.), and the diameter of the retrieved cells ranged from 0.1 and 0.4 μm. Taking this diameter as a starting point and considering each cell in the shape of a sphere, it was possible to calculate the bacterial biovolume, and then, using the following equation biomass pgC cell^−1^ = 0.2*V^0.72^ (Moran et al., 2015), the average biovolume was converted into biomass; average cell biomass times cell abundance resulted in total biomasses ranging between 1.58 ·10^2^ and 4.77 · 10^3^ pgC mL^−1^ being higher in coastal stations S, M and in the harbor area.

### Computational cytometry

As stated above, the automated process produces a dataset useful for computational cytometry. Each gated cell is described by a set of measurements consisting of the height and area of the emission peak for each collector filter and representing a unique optical signature for each particle (Rubbens and Props, 2021). Based on this information it is possible to (i) identify groups of particles with similar characteristics, (ii) calculate alpha diversity, and (iii) compare communities (beta diversity).

**Figure 5:**
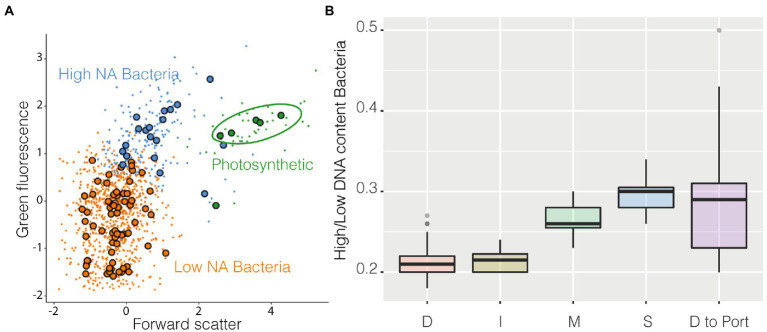
FlowSOM results, panel **(A)** is a resume cytogram that shows three of the groups identified by the algorithm (metaclusters), photosynthetic prokaryotes in green, high NA content prokaryotes in blue, and low NA content bacteria in orange, large dots represent the geometrical center of each of the 100 clusters on which metaclusters are built; on panel **(B)** is shown as boxplot the ratio High/Low of all the samples belonging to the different stations and the final transect between station D and the harbor.

Among the groups identified by the algorithm as metaclusters, three were easily recognizable as known classical populations: High NA content prokaryotes, Low NA content Bacteria, and cyanobacteria (Figure 5A). Cyanobacteria (Supplementary Figure S6) were more abundant in coastal stations compared with station D and decreased in abundance near the harbor. High NA content bacteria could be caused by the presence of more RNA copies and/or dividing cells, and it has been suggested to be a proxy of more active cells (Li et al., 1995). The ratio High/Low has often been used as a possible indicator of the degree of community activity (a more active community has a higher ratio). These higher activity bacteria were more abundant at coastal stations compared with station D and I. The ratio also increased in the samples closer to the harbor (Figure 5B).

**Figure 6:**
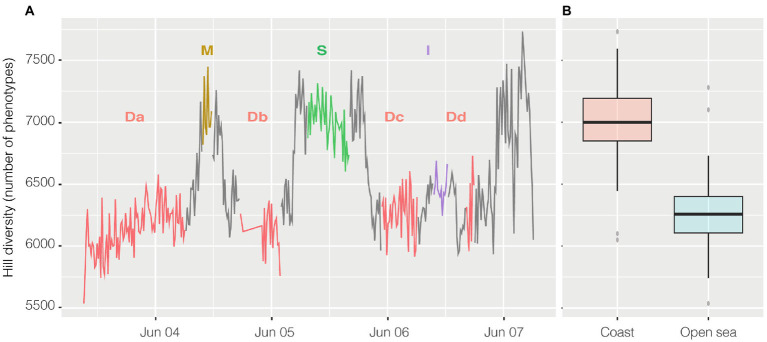
Hill Diversity, panel **(A)** shows the variation of alpha diversity along the entire cruise. On the x axis time is plotted whereas on the y axis the values of Hill diversity are expressed as “number of phenotypes” i.e. the number of bins collecting cells with shared characteristics. The same data are visualized in panel **(B)** divided in coastal samples (from 41° of latitude to higher latitudes) and open ocean samples (from 41° to lower latitudes). Transits between stations reported on the graph are in grey.

Alpha diversity was calculated with the R package PhenoFlow as Hill diversity (Props et al., 2016). Hill diversity could be interpreted as ‘effective number of species’ and, in this case, similarly to the Simpson index, the calculated value represents the number of equally abundant species required to generate an identical diversity as that of the observed microbial community (Jost, 2006). Here “species” should be intended as phenotypes, i.e., each one of the bins collecting cells with similar fluorescence and scatter properties. Briefly, each cytogram was divided into a grid of 128 × 128 bins and the particles density in each bin was the basis for diversity calculations. Hill diversity index D2 (Figure 6A) ranged from 5,742 to 7,731, and excluding the harbor, had a strong inverse relationship with abundance (*n* = 356, *R* = −0.65 and *p* < 0.001) and, actually, had a higher value in the coastal area above 41° of latitude (Figure 6B), except for the harbor where went back down. Alpha diversity had a strong inverse relationship both with salinity (*n* = 356, *R* = −0.67, *p* < 0.001) and conductivity (*n* = 356, *R* = −0.63, *p* < 0.001) and less so with temperature (*n* = 363, *R* = −0.42, *p* < 0.001).

### Possible problems, pitfalls, artifacts and suggested solutions

Our results highlight that the method presented here is robust and very useful, however, some problems/risks should be taken in account. They are listed below from least to most important.

Use of glass bottles: Considering the use of corrosive, toxic and teratogen chemicals, it is worth to stress the importance of a careful setting of the system. Special attention should be paid to the protection of the SGI amber bottle. If the cleaning solution is closed with only parafilm, remember to fix it as much as possible and away from the computer and other electrical components. To prevent incidents, it is possible to use special lids.

Storm and spills: Although we were able to demonstrate that the method works well during a storm, there are 3 issues to be aware of: (1) the liquid inside the incubation chamber could spill out with strong movements (2) the collection tubes of the cleaning bottles could be above of the liquid level at the time they operate, (3) the pipe of the inlet seawater pump could move and not pour water into the collector container where the sample tubes are placed. There are no possible solutions for an internal spill of the incubation chamber and it is important to double check the data to ensure that they are biologically sound. On the other hand, there is no risk for the operator since the spill would occur inside the chamber. About possible problems with the bottles, it is important to periodically check during a storm that the tubes are sitting near the bottom of the bottle and tape them in place. To keep collecting water in stormy conditions, try using a larger collection container (if there is a strong flow that ensures the continuous renewal of water) or, if a falcon is used as the collector, a “leaky” half funnel might be useful for intercept the moving stream of water (Supplementary Figure S1).

Unfiltered water and clogging of the tubes: Water collected from a Niskin is normally prefiltered through a 20 μm mesh, this step is absent with automatic sampling. A bloom of large phytoplankton or, in general, high turbidity levels may cause clogging in both the flow cytometer and the OC-300. A possible solution for this situation is to add a mesh to the faucet that provides water, cleaning it every day. This could be important especially in coastal areas but not in clear water oceanic areas. The mesh should be rinsed daily.

Clogging of the valve: The valve where the syringe is mounted could get clogged during the cruise. If this happens, less liquid is incorporated by the system and irregular bubbles appear. The fastest way to solve this problem is to disassemble the valve, clean it (with compressed air through each hole, one night in ultrapure water, and again a round of compressed air in each hole) and put it back in place. If the machine is not new, it is probably a good idea to do it anyway before a cruise.

**Box 1**

**Sampling**

Seawater is collected from a Niskin bottle and prefiltered through a mesh of 20 μm.

**Fixation**

1800 μL of seawater +200 μL of a mix of paraformaldehyde 1% and glutaraldehyde 0.05%.

**Stain**

1 μL of SYBR Green I for each 100 μL of fixed sample, incubation of 10 min in the dark at RT.

**Flow cytometry**

The sample it is excited by a blue laser and the light emission collected by a bandpass 530/30 nm filter, it runs for 2 minutes at low speed (14 μL min^−1^).

## Discussion

Automated flow cytometry has the obvious advantage of improving sampling resolution; for example, in the present study, the regular sampling plan for the MIAU oceanographic cruise involved 4 surface samples, one for each station, while with the OC-300 we sampled surface waters 366 times, thus increasing the number of samples by more than 90 times. In terms of economics, automating the process allows for better use of sailing time, even during a storm, and the effort to set up the system on the first day of cruising is fully balanced by the small amount of work required during the rest of the days, and by the number of samples taken. Considering that for each sample point we can extract information about abundance, cellular size, biomass, diversity and (phenotypic) community composition, the dataset produced is extremely useful.

Data obtained with the automatic sampler were compared with samples analyzed with the usual protocol (Box 1) which was applied to samples belonging to water from the inlet pump (*n* = 10), this comparison shown that OC-300 tend to give slightly higher values showing a moderate direct relationship with data from the standard protocol.

Overall, the results here presented are sound, they appear to have an ecological meaning and they are in agreement with the results found with the normal method. There is a clear geographical structure for all the studied parameters, probably determined by a change in the main water mass around latitude 41° (Supplementary Figure S7), which is associated with a change in salinity. Compared to stations D and I, the coastal stations M and S presented lower abundance (perhaps counterintuitively), higher diversity, and an apparently more active prokaryotic community. Interestingly, the area near the Barcelona harbor has a very different pattern compared with the coastal stations. When we entered the port, in the last hour of sailing, prokaryotic abundance peaked reaching the highest values of the entire cruise, whereas diversity and average cell size went down abruptly. The ratio High/Low NA content prokaryotes was also highest in the port area whereas photosynthetic microbes decreased remarkably. The effect of the harbor structure over nearby coastal communities is seldom inspected, yet thanks to the automatic sampling we were able to obtain hints of the microbial communities inside the man-made structure.

The growth rate observed during the diel cycles, of 0.04 ± 0.01, is similar to that observed at the same station in 1995, which was of 0.07 ± 0.03 (Pedrós-Alió et al., 1999). This low value could be explained by biotic interactions (grazing and/or viral lysis) or by the lack of sufficient nutrients for the growth of the population. We have not measured either of these variables in continuum.

Most aquatic microbial studies associate flow cytometry with abundance values only, but the huge amount of data produced (for each of the 366 sampling points there were 10,000 cells subsampled and for each cell there were 6 difference characteristics, scatter or fluorescences, collected) highlight the power of flow cytometry as a high-throughput technique. Handling of this type of dataset could have been difficult and tedious in the past but luckily, paralleling what has happened with DNA sequencing, several bioinformatic tools have been developed recently under the name of “computational cytometry.” Applying these techniques to the dataset allowed to identify the classical groups (High and Low NA content prokaryotes, photosynthetic prokaryotes) in a standardized way (i.e., not depending on the operator decisions), which results on a decrease of the variability between operators and between studies when a subpopulation is defined. The ratio High/Low suggests that more active cells were present in the coastal area (Station M and S), although it is worth to mention that the number of chromosome copies, as well as their size, are strain specific, and hence, it could be that an inactive or not dividing cell with a high number of chromosomes is stained with the same intensity, and so occupies the same position in a cytogram, as a small very active cell which has more mRNA copies or is actively synthesizing DNA (Cichocki et al., 2020).

Moreover, it was also obtained an estimation of diversity (i.e., phenotypic diversity) based on flow cytometry data, which was found to correlate well with the 16S rRNA gene diversity in several environments including the ocean (García et al., 2015). Here the estimated diversity values were not random, they were higher in the coastal area than in the open sea, as there were probably more niches and resources to exploit compared to the open ocean, but they dropped when approaching the harbor likely because of the presence of a more polluted environment. It is important to stress the relevance and novelty of determining microbial diversity at this unprecedent scale. The diversity of a similar transect was analyzed by Pommier and colleagues in 2010 (Pommier et al., 2010), based on pyrosequencing of the 16S rRNA gene. These authors found that station D and M had lower values of diversity compared with more coastal samples. At that time, they observed, as we did, a lower value of diversity in station D.

Although we presented here only data for prokaryotes, and given that FC appears well suited to analyze a whole set of microbial populations (Gasol and Morán, 2015), this method could easily be applied to phytoplankton, and, with some adjustments, to heterotrophic flagellates and viruses, opening the possibility of reaching a comprehensive picture of the environment at a fine scale, collecting information about interactions such as grazing, infection and carbon flux in general.

In addition, the OC-300 system allows monitoring of the prokaryote abundance in real time. Considering the detection of the abrupt change in abundance around 41° of latitude it could be used in the future, in association with physical parameter measurements, as an easy way to detect changes in water masses defined for their different microbial communities. The potential of this methodology to detect rapid environmental changes and microenvironments along the track is huge and yet unexplored.

Automation of flow cytometry presents some limitations, in the specific the main mechanical issue we encountered was the clogging of the valve and its relative cleaning which, although fixable, involves the loss of one day of sampling, as we experienced in a posterior cruise. It is also important to stress that the automated sampling is limited to the ocean surface only since, at the moment, there is no technology available to pump waters continuously from other depths. Despite these limitations, overall we found the automatic sampler to be very useful and a clear improvement to the toolbox of oceanographic cruises. In particular, this methodology is especially adequate to detect temporal trends, e.g., between day and night, or spatial ones between crossed water masses, e.g., the change in abundances along the cruise in station D could have easily been missed with standard sampling.

## Data availability statement

The datasets presented in this study can be found in online repositories. The name of the repository and accession link can be found at: SEANOE; https://doi.org/10.17882/90671.

## Author contributions

MP performed all the tasks during the cruise and the analyzes of the dataset. MP and JG wrote the manuscript. All authors contributed to the article and approved the submitted version.

## Funding

The OC-300 and the cruise were funded by project MIAU (RTI2018-101025-B-I00) from the Spanish Ministry of Science and Innovation. We acknowledge project PID2021-125469NB-C31 form the same Ministry, and the generic support by the ‘Severo Ochoa Centre of Excellence’ accreditation (CEX2019-000928-S).

## Conflict of interest

The authors declare that the research was conducted in the absence of any commercial or financial relationships that could be construed as a potential conflict of interest.

## Publisher’s note

All claims expressed in this article are solely those of the authors and do not necessarily represent those of their affiliated organizations, or those of the publisher, the editors and the reviewers. Any product that may be evaluated in this article, or claim that may be made by its manufacturer, is not guaranteed or endorsed by the publisher.
