# Supplementary material for: Automated flow cytometry as a tool to obtain a fine-grain picture of marine prokaryote community structure along an entire oceanographic cruise
Source: Front Microbiol. 2023 Jan 6;13:1064112. doi: 10.3389/fmicb.2022.1064112 (PMC9853387; doi:10.3389/fmicb.2022.1064112)
Supplement: Supplementary file 1 [file Data_Sheet_1.docx]

Supplementary material

Figure S1: Sketch of the sample system including the falcon and the half funnel, in red is highlighted a hypothetical maximum of the range of movement of the water flow during a storm compared with a normal weather condition (in green) when the flow of water doesn’t change position.

Figure S2: Effects of the spillover of SGI into the red channel (FL3). In panel A it is shown the fluorescence in green of prokaryotes stained with SGI. The same population is plotted in a cytogram of green (FL1) versus red (FL3) in panel B. It is possible to see that SGI has a spillover of the signal into the red channel which lead to the visualization of the heterotrophic prokaryotes population in a diagonal position, nevertheless the autotrophic population (red dotted ellipse) has a stronger red fluorescence component which make the population pop up from the bulk of prokaryotes cells.

Figure S3: Prokaryotic abundance through time, the storm is highlighted by the light blue square.

Figure S4: Measures of abundance along time (pink, left axis) from a posterior cruise compared with the movement of the ship (roll angle, in grey, unity on the right axis).

Figure S5: Map of the distribution of the relative size (i.e. median value of forward light scatter) along the cruise.

Figure S6: Metacluster of photosynthetic bacteria as identified by FlowSOM along the entire cruise.

Figure S7: (a) Map of Salinity (b) Map of Diversity (c) Map of the ratio High/Low NA content bacteria

Supplemental text

Command line to quickly change the names in python:

for i in 2021-0* ; do while read e ;do cp ${i}/${e} /path_to_your_output_folder/${i}_${e} ; done < ../names.txt ; done
