# Supplementary figures and images for: Automated flow cytometry as a tool to obtain a fine-grain picture of marine prokaryote community structure along an entire oceanographic cruise
Source: Front Microbiol. 2023 Jan 6;13:1064112. doi: 10.3389/fmicb.2022.1064112 (PMC9853387; doi:10.3389/fmicb.2022.1064112)

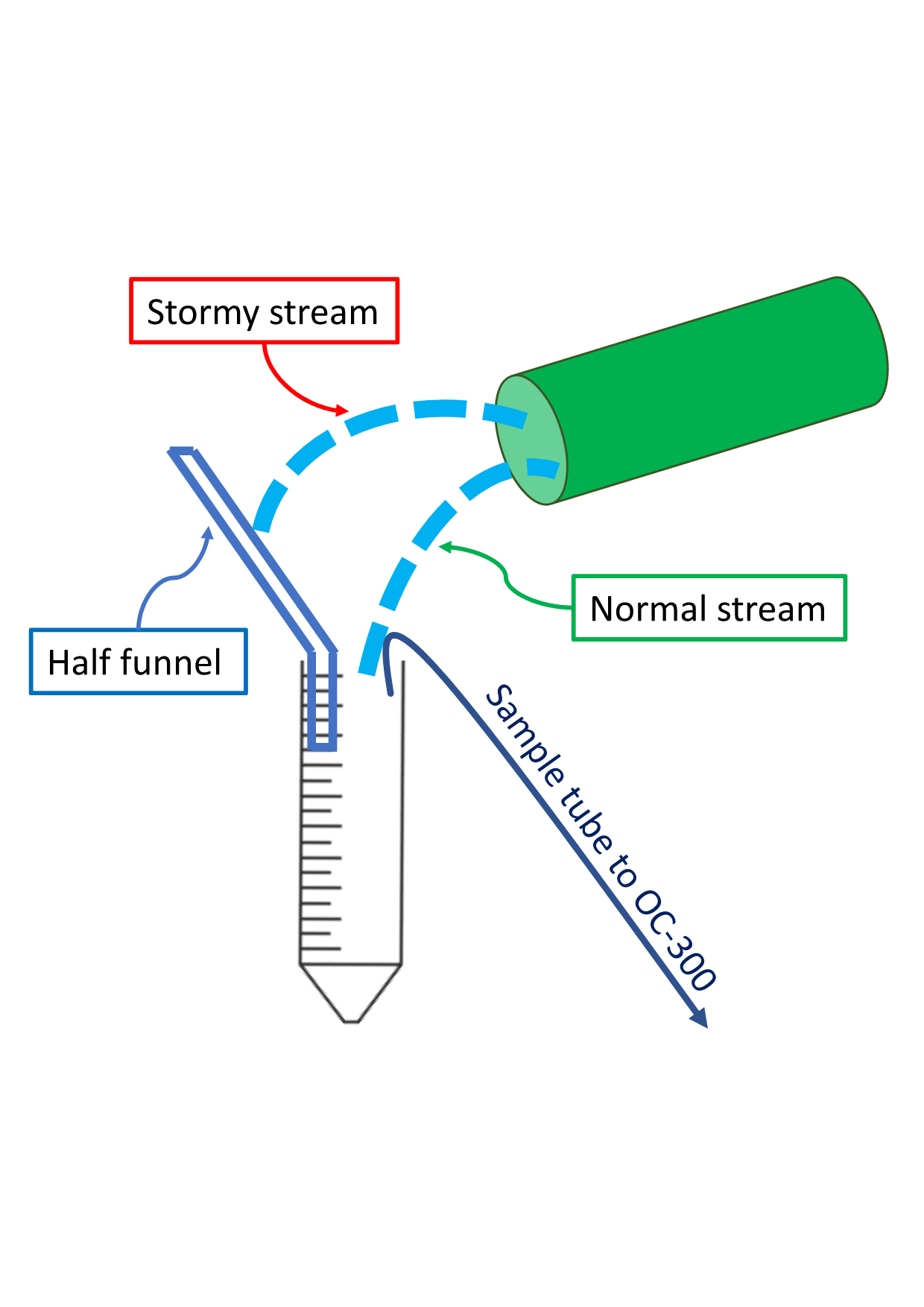

Supplement: Supplementary file 2 [file Image_1.TIFF]

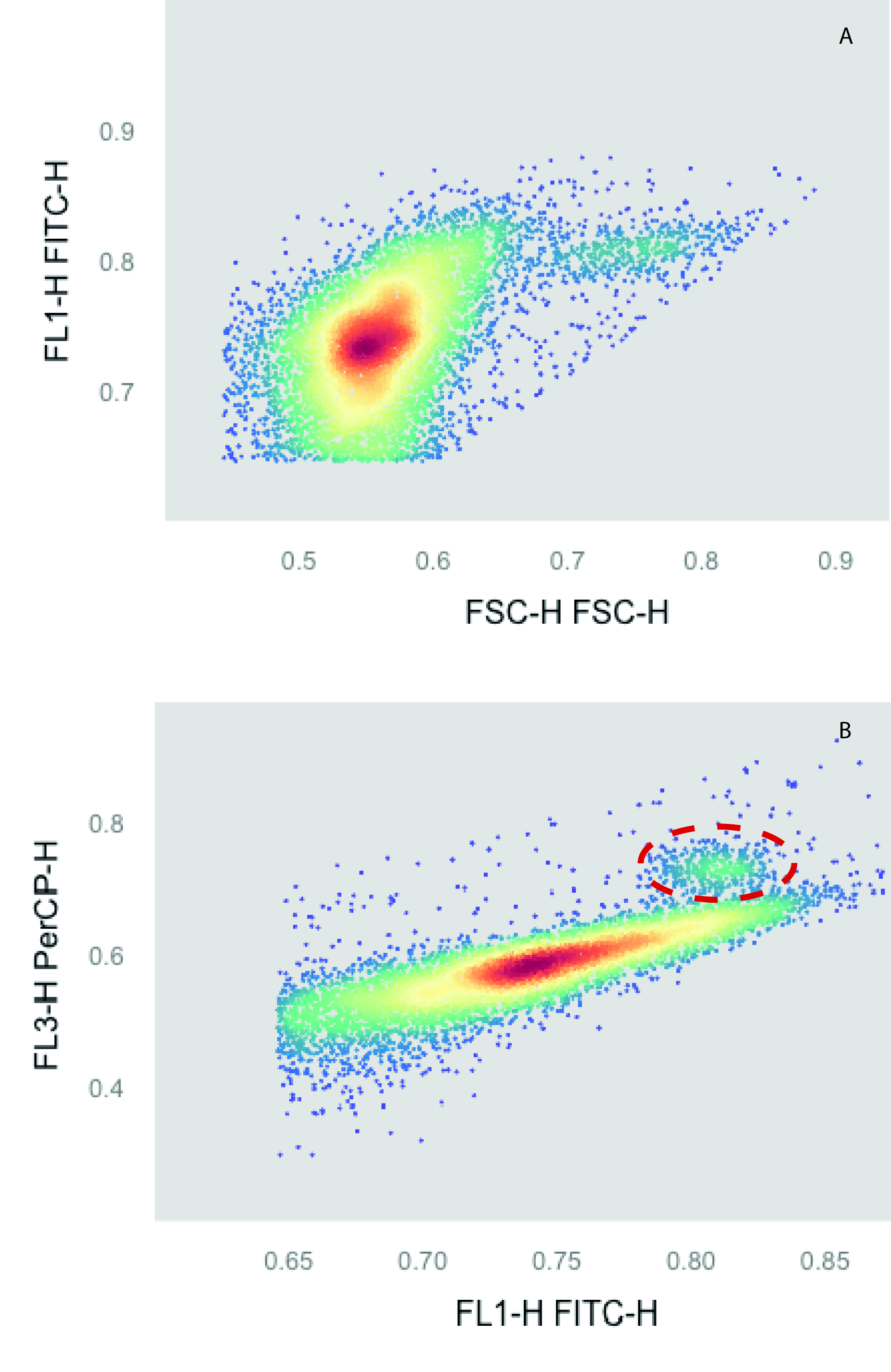

Supplement: Supplementary file 3 [file Image_2.TIF]

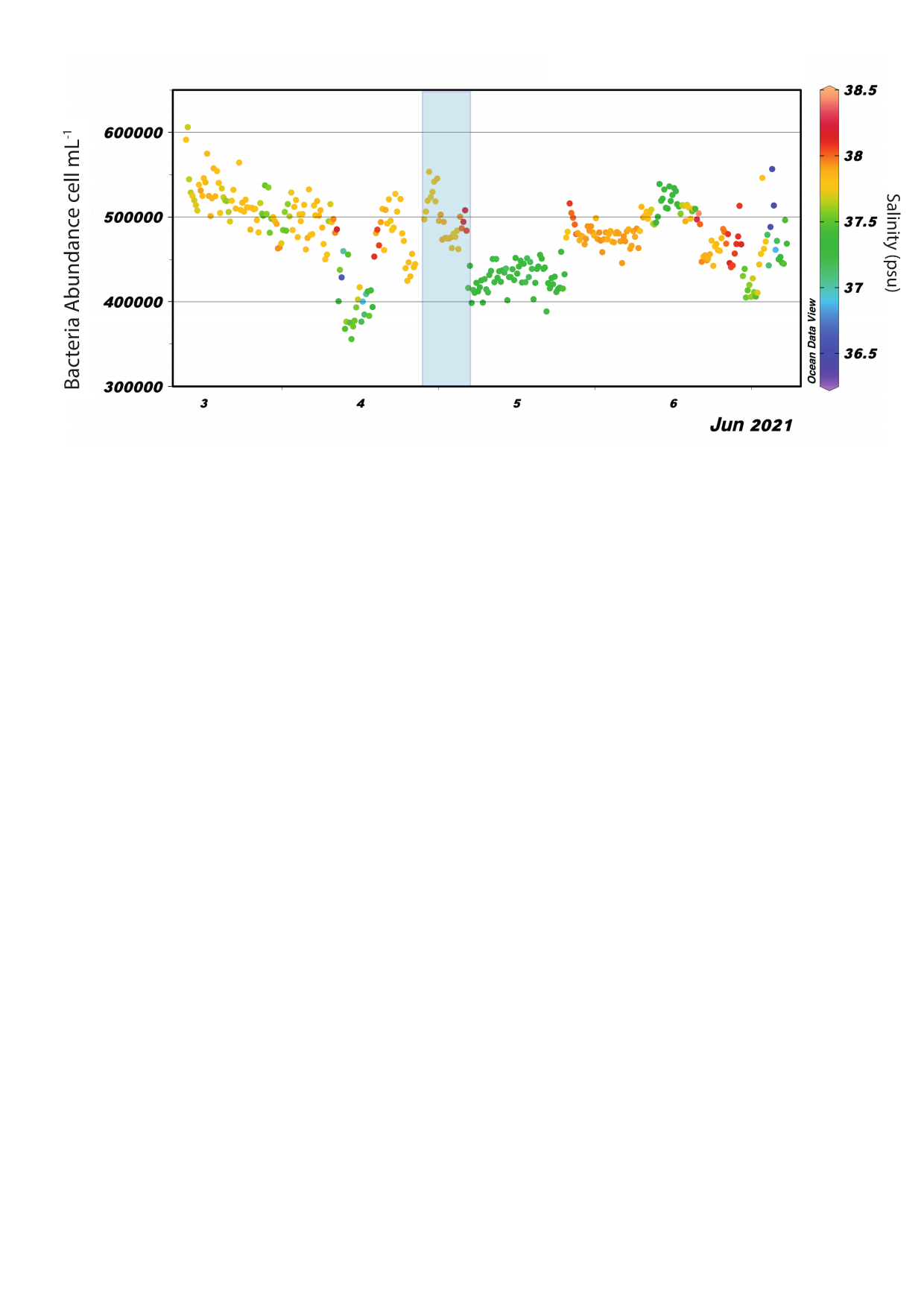

Supplement: Supplementary file 4 [file Image_3.TIFF]

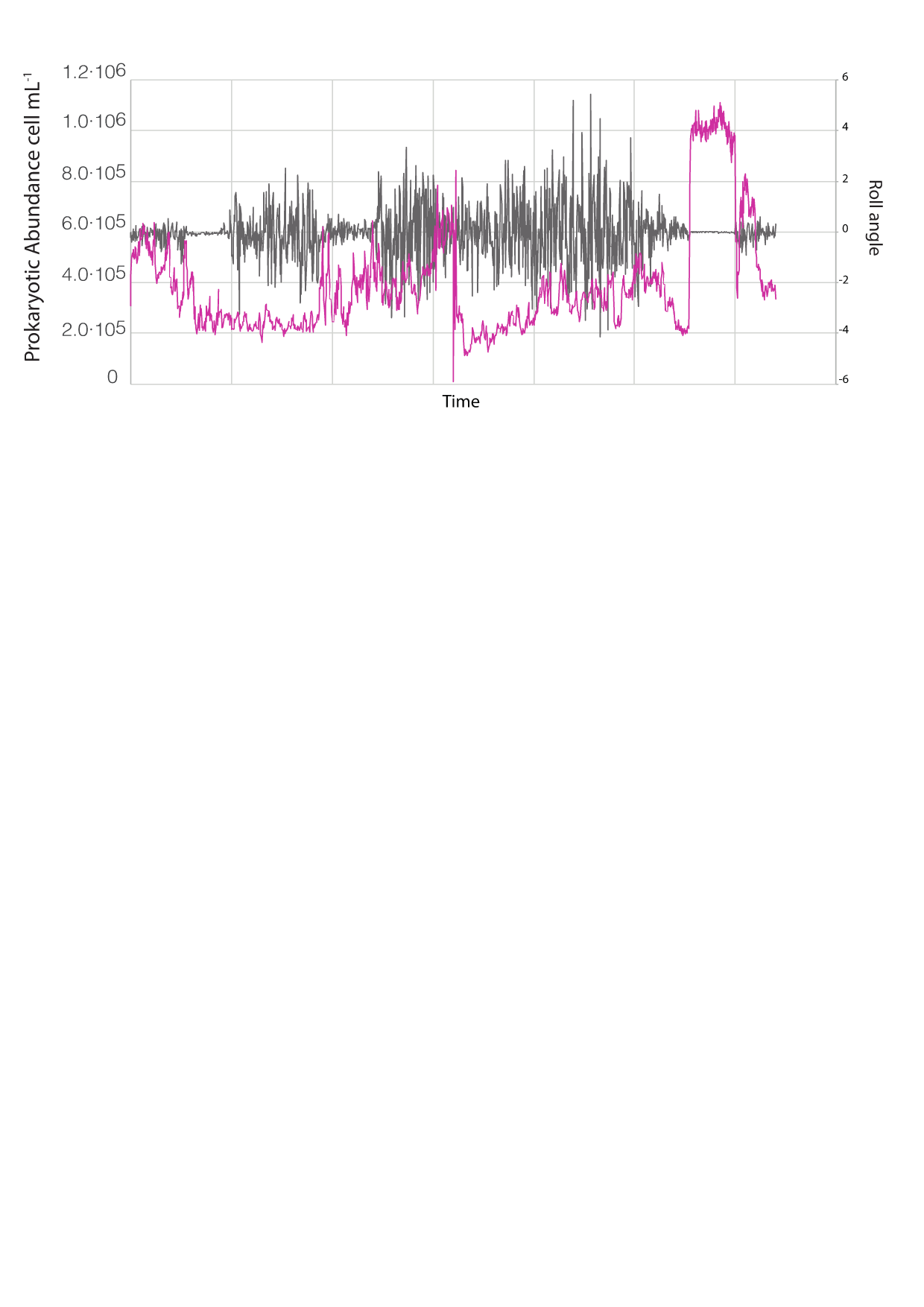

Supplement: Supplementary file 5 [file Image_4.TIFF]

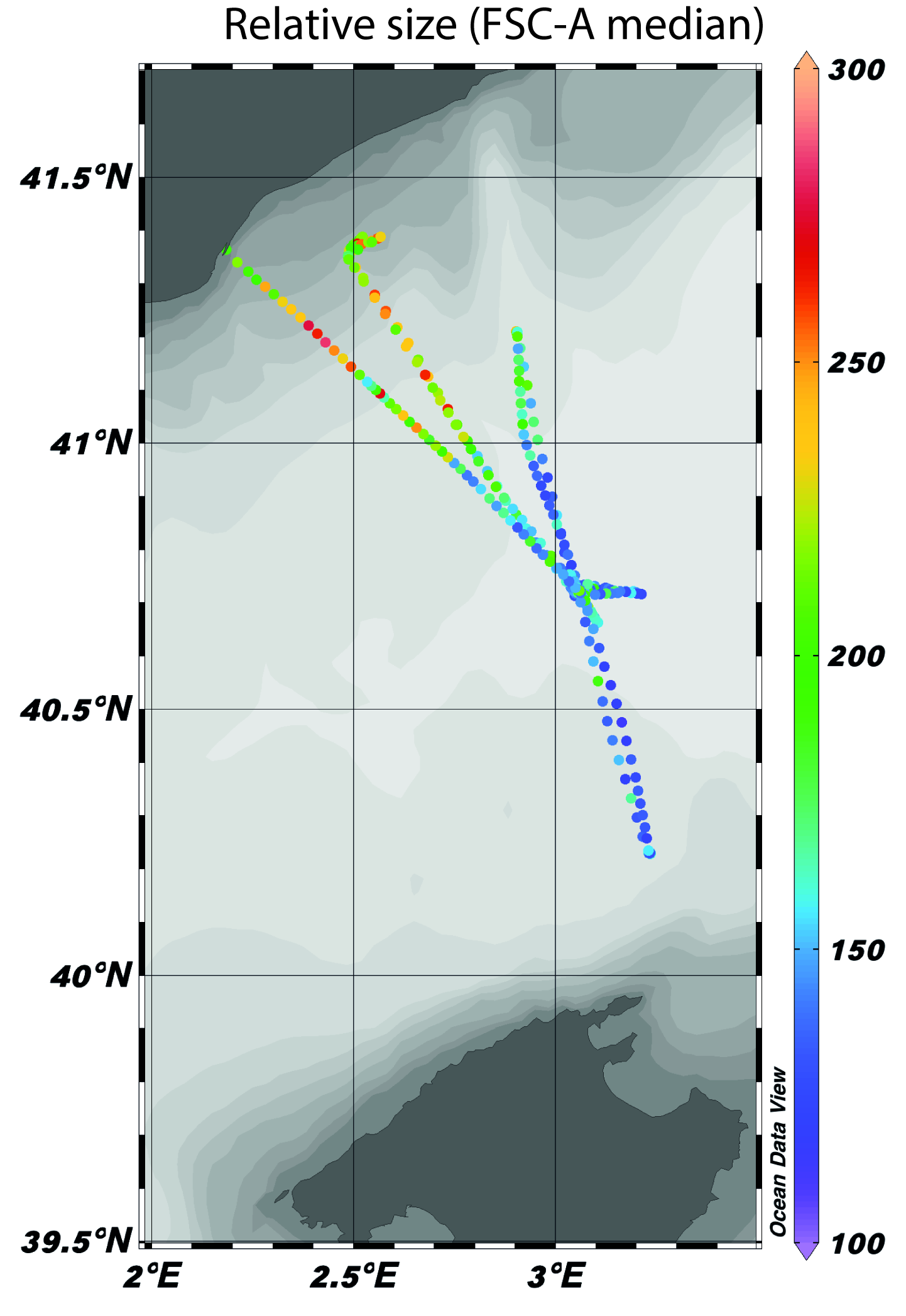

Supplement: Supplementary file 6 [file Image_5.TIF]

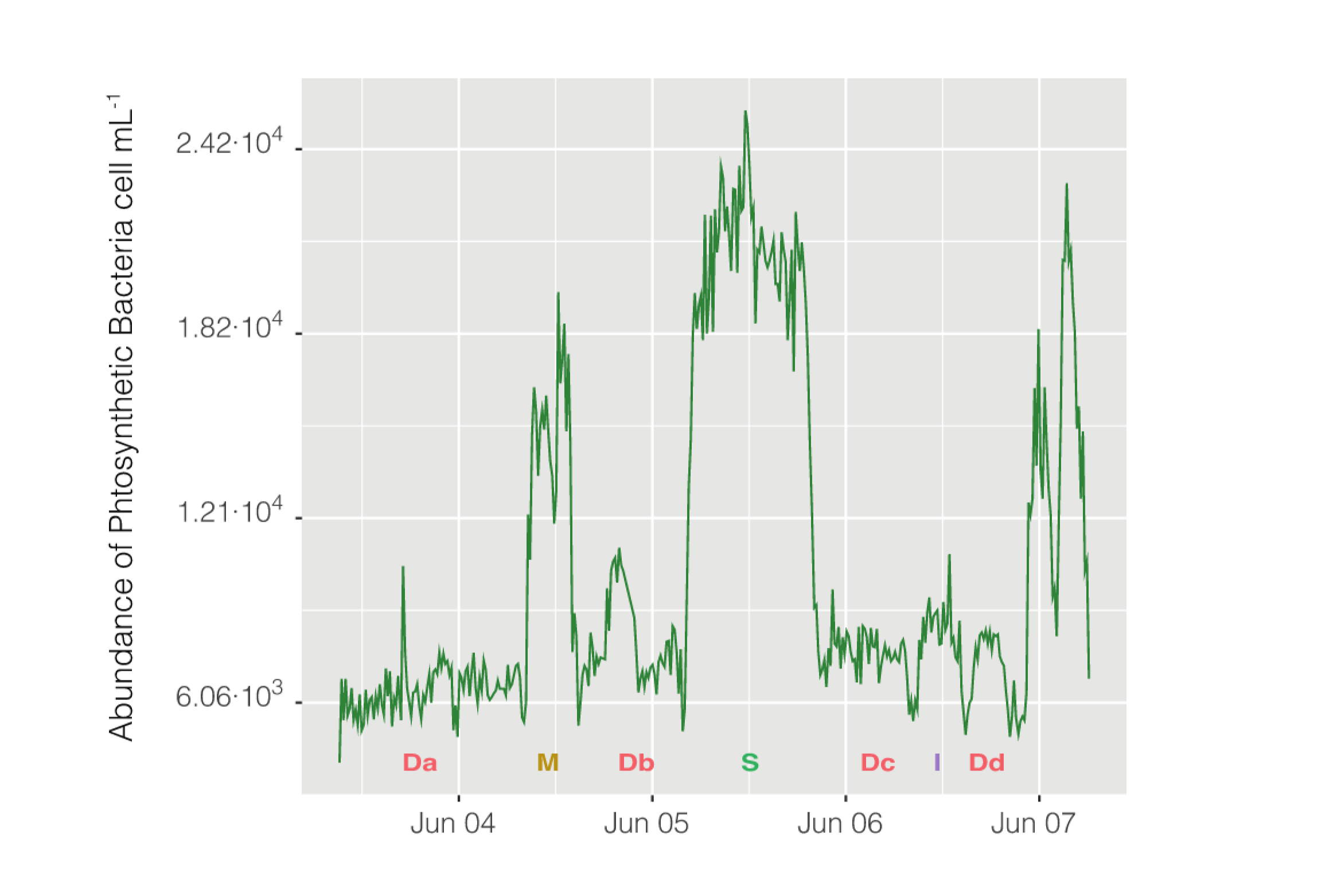

Supplement: Supplementary file 7 [file Image_6.TIF]

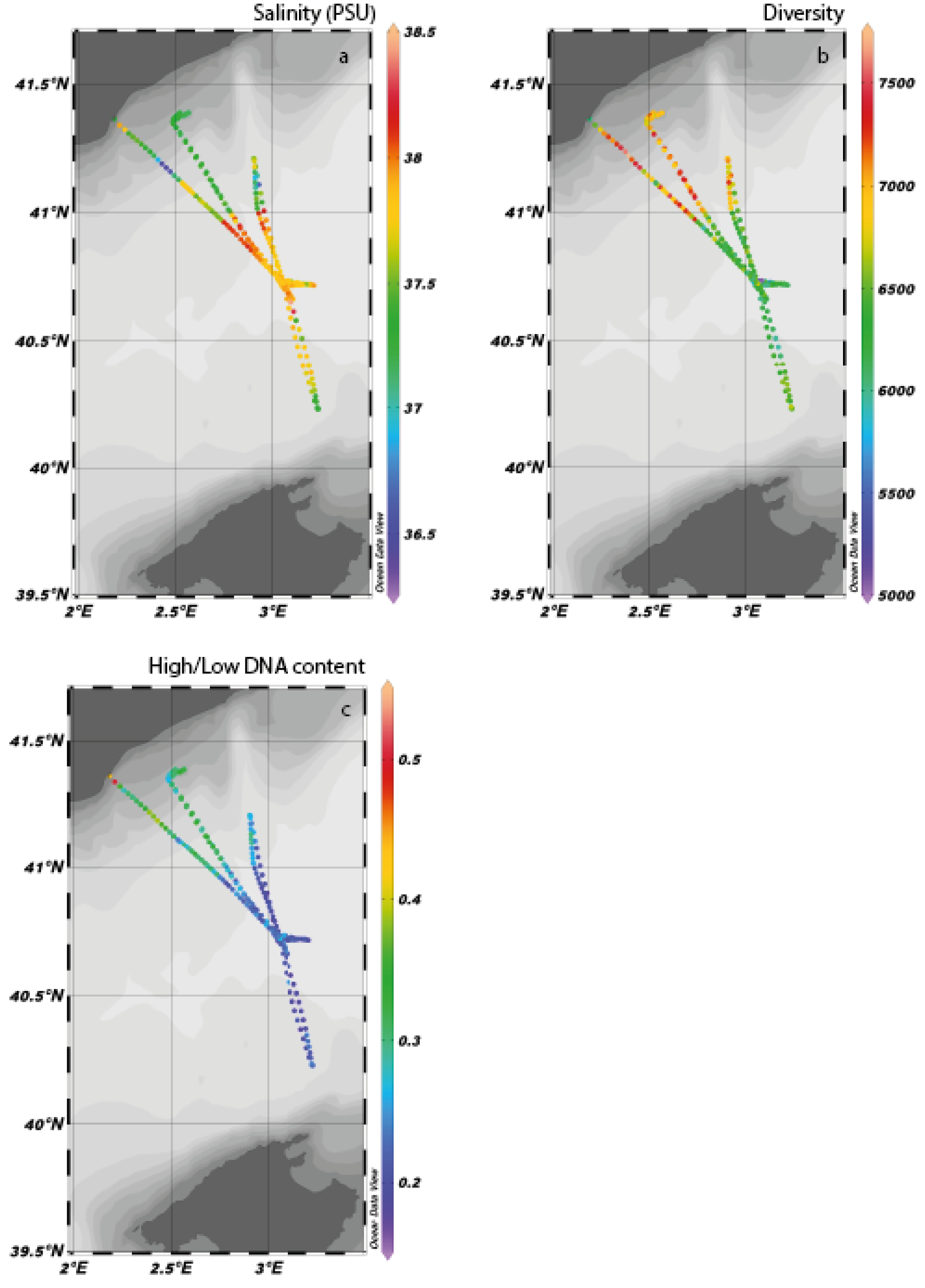

Supplement: Supplementary file 8 [file Image_7.TIF]
